# Field trial on glucose-induced insulin and metabolite responses in Estonian Holstein and Estonian Red dairy cows in two herds

**DOI:** 10.1186/1751-0147-52-4

**Published:** 2010-01-20

**Authors:** Hanno Jaakson, Katri Ling, Jaak Samarütel, Aire Ilves, Tanel Kaart, Olav Kärt

**Affiliations:** 1 Institute of Veterinary Medicine and Animal Sciences, Estonian University of Life Sciences, 1 Kreutzwaldi St, 51006, Tartu, Estonia

## Abstract

**Background:**

Insulin secretion and tissue sensitivity to insulin is considered to be one of the factors controlling lipid metabolism *post partum*. The objective of this study was to compare glucose-induced blood insulin and metabolite responses in Estonian Holstein (EH, n = 14) and Estonian Red (ER, n = 14) cows.

**Methods:**

The study was carried out using the glucose tolerance test (GTT) performed at 31 ± 1.9 days *post partum* during negative energy balance. Blood samples were obtained at -15, -5, 5, 10, 20, 30, 40, 50 and 60 min relative to infusion of 0.15 g/kg BW glucose and analysed for glucose, insulin, triglycerides (TG), non-esterified fatty acids (NEFA), cholesterol and β-hydroxybutyrate (BHB). Applying the MIXED Procedure with the SAS System the basal concentration of cholesterol, and basal concentration and concentrations at post-infusion time points for other metabolites, area under the curve (AUC) for glucose and insulin, clearance rate (CR) for glucose, and maximum increase from basal concentration for glucose and insulin were compared between breeds.

**Results:**

There was a breed effect on blood NEFA (*P *< 0.05) and a time effect on all metabolites concentration (*P *< 0.01). The following differences were observed in EH compared to ER: lower blood insulin concentration 5 min after glucose infusion (*P *< 0.05), higher glucose concentration 20 (*P *< 0.01) and 30 min (*P *< 0.05) after infusion, and higher NEFA concentration before (*P *< 0.01) and 5 min after infusion (P < 0.05). Blood TG concentration in ER remained stable, while in EH there was a decrease from the basal level to the 40^th ^min nadir (*P *< 0.01), followed by an increase to the 60^th ^min postinfusion (*P *< 0.01).

**Conclusion:**

Our results imply that glucose-induced changes in insulin concentration and metabolite responses to insulin differ between EH and ER dairy cows.

## Background

Selection for higher milk production has been associated with changes in cows' metabolism, especially the capability to partition more energy into milk [1] and less into body reserves, and with enforced *post partum *lipid mobilization [2]. These changes have increased the incidence of a range of metabolic disorders such as fatty liver/ketosis complex and displaced abomasum [3], reduced cow fertility [4] and immunity [5]. Identifying the mechanisms that control lipid metabolism could help to develop strategies to improve cows' health and welfare, as well as fertility.

In dairy cows, insulin and tissue sensitivity to insulin, which can be studied using an intravenous glucose infusion method, known as the glucose tolerance test (GTT), play a key role in the regulation of *post partum *nutrient partitioning and lipid mobilization [6]. At the beginning of lactation reduced blood glucose concentration is associated with relatively low blood insulin levels [7-9]. In addition, during the period of negative energy balance (NEB), lasting up to 70 days *post partum *[10], dairy cows show a weaker glucose-induced insulin response [11,12] and reduced tissue sensitivity to insulin [13,14] compared to the *pre partum *period. High producing cows have lower *post partum *blood insulin concentrations compared to less productive animals [15]. In addition, Holstein cows express stronger lipolytic responses to energy deficiency compared to Jersey cows [16] and have lower blood insulin concentrations and weaker insulin responses to glucose injection compared to beef cows [17,18]. These findings suggest that partitioning of energy between the tissues and milk, intensity of lipolysis, blood insulin level and insulin response to glucose injection could be related to cow breed.

In Estonia the average 305-day milk yield in 2008 was 7,577 kg in Estonian Holstein (EH) and 6,855 kg in Estonian Red (ER) cows [19]. We hypothesize that EH cows, as higher producers, may have a weaker insulin response and reduced sensitivity to antlipolytic and lipogenic effects of insulin compared to ER cows. Therefore, the aim of this study was to compare glucose-induced blood insulin and metabolite responses during the period of NEB in EH and ER cows.

## Methods

### Farms, animals and experimental design

The study was carried out on two similar management and high production level dairy farms during the housed period, in March and April 2005 on farm A and in February 2008 on farm B, on 28 clinically healthy multiparous (2^nd ^to 5^th ^parity) cows. Two experimental groups were formed as follows: EH (n = 14; six cows from farm A and eight from farm B) and ER (n = 14; eight cows from farm A and six from farm B). Cows' body weights (BW) were 674 ± 23 kg for EH and 583 ± 22 kg for ER. On both farms cows were housed in tie stall barns and offered a total mixed ration (TMR) *ad libitum *twice a day, around 09.00 and 18.00. TMR samples were collected once a week throughout the whole experimental period and pooled. Feed values of TMR were calculated on the basis of the chemical composition of the ingredients, determined according to AOAC [20] methods in the pooled samples. TMR offered to the 15 to 150 days in milk feeding group, including the experimental cows, provided 11.4 MJ metabolizable energy (ME) and 96.6 g metabolizable protein (MP) per kg dry matter (DM) on farm A, and 12.0 MJ ME and 105.5 g MP per kg DM on farm B (Table 1). Approximate DM intake (DMI) of the feeding group was about 22 kg/d per cow on both farms and this was evaluated using a method appropriate for field conditions, as follows. On both farms, during the whole experimental period, the amount of TMR was weighed and on the basis of visual every day assessment of feed refusal, average DMI of the feeding group including experimental cows during the glucose tolerance test (GTT) was estimated per week. Cows were milked thrice a day; approximately from 05.00 to 08.00, from 12.30 to 15.00 and from 20.00 to 23.00 on both farms. Average 305-day energy corrected milk (ECM) yield from the previous lactation in the experimental groups was 8,999 ± 319 kg for EH and 8,253 ± 287 kg for ER cows. ECM yields during the experiment, calculated as the mean of day before and day after GTT, were 36.8 ± 2.6 kg/d (EH) and 38.0 ± 2.5 kg/d (ER); mean milk constituents, determined by an infrared spectrometry (MilkoScan 4300, Foss Electric A/S, Slangerupgade 69, 3400 Hillerød, Denmark), were 4.56 ± 0.25% and 4.66 ± 0.25% for fat, and 3.11 ± 0.11% and 3.64 ± 0.11% for protein in EH and ER respectively. Cow body condition scores (BCS) were evaluated by the same observer according to the method of Edmonson et al. [21] at calving and weekly up to the tenth week *post partum*.

**Table 1:** Ingredients and chemical composition of TMR on farms A and B.

	Farm A	Farm B
*Ingredients (% DM)*		

Grass silage	37.31	20.29

Corn silage	-	15.91

Hay	2.44	3.67

Barley meal	14.08	-

Corn meal	23.23	9.64

Wheat meal	-	9.62

Rape cake	18.92	25.87

Soyabean meal	2.58	7.56

Palm oil	-	2.24

Brewers grains	-	4.93

Mineral feed	0.36	0.55

Limestone	0.24	0.42

Salt	0.57	0.42

Soda	-	0.85

*Chemical composition*		

Dry matter (%)	53.4	56.2

In dry matter		

NDF (g/kg)	314.2	372.0

ADF (g/kg)	213.9	227.8

Crude protein (g/kg)	164.5	197.5

Crude fat (g/kg)	57.9	70.2

Ca (g/kg)	8.6	6.2

P (g/kg)	5.1	5.3

ME (MJ/kg)	11.4	12.0

MP (g/kg)	96.6	105.5

The European Council Directive regarding the protection of animals and the Estonian Animal Protection Act have been complied with in this experiment. The study has been approved by the committee of animal experimentation of the Estonian Ministry of Agriculture.

### Glucose tolerance test

The GTT was carried out according to the protocol described by Holtenius et al. [22] on average 31 ± 1.9 days *post partum* after the morning milking at around 10.00, on two to four cows on the same day. Feed was withdrawn 60 min before and during the GTT. A catheter (12G, Lenght 80 mm; Jørgen Kruuse A/S, Havretoften 4, DK-5550 Langeskov, Denmark) was inserted into the jugular vein and fixed to the skin 30 min before the test. The catheter was filled with Li-heparin (LEO Pharma, Longwick Road, Princes Risborough, Buckinghamshire, HP27 9RR, United Kingdom) until the start of blood sampling and between samplings to avoid clotting. After infusion of 0.15 g/kg BW glucose (40%-solution; Inj. Glucosi 40%; Vetoquinol Biowet Sp. z o.o. ul. Kos. Gdyñskich 13-14 66-400, Gorzów Wlkp., Poland), for approximately 4 min, the tubing and catheter were flushed with normal saline (Sodium Chloride 0.9% I.V. Inf.; B. Braun Melsungen AG, Carl-Braun-Straße 1, 34212 Melsungen, Germany). Discarding the first portion, blood samples were collected into vacuum tubes with Li-heparin (BD Vacutainer Systems, NH 170 I.U., Plymouth, United Kingdom) at the following times: -15, -5, 5, 10, 20, 30, 40, 50 and 60 min relative to the start of infusion. Plasma was separated by centrifugation (5000×g, 15 min) immediately after sampling and kept at -24°C until analysed.

### Analyses

Concentrations of metabolites in plasma were analyzed spectrophotometrically (Helios β; Unicam Ltd., PO Box 206, York St., Cambridge, CB1 2ST, United Kingdom) using standard test kits (Human Gesellschaft für Biochemica und Diagonostica GmbH, Max-Planck-Ring 21 - D-65205 Wiesbaden, Germany) for glucose (mmol/l), triglycerides (TG; mmol/l) and cholesterol (mg/dl); and test kits from Randox Laboratories Ltd. (Ardmore, Diamond Road, Crumlin, Co. Antrim, United Kingdom, BT29 4QY) for NEFA (μmol/l) and β-hydroxybutyrate (BHB; mmol/l). Blood insulin concentration (μIU/ml) was measured radio-immunologically (Wallac 1470 Wizard Gamma Counter; Perkin Elmer Life and Analytical Sciences, Inc., 940 Winter Street, Waltham, Massachusetts 02451 USA) using ^125^I radioimmunoassay test kits (Coat-A-Count Insulin) from Siemens Medical Solutions Diagnostics (5210 Pacific Concourse Drive. Los Angeles, CA 90045-6900, USA). The inter- and intra-assay coefficients of variation of the methods were below 6%.

### Calculations and statistical analyses

The following measurements in EH and ER cows were calculated and/or compared: BCS at calving and at the time of the GTT, and BCS loss from calving to GTT; cows BW and milk yield during the GTT; basal concentration of metabolites at the time of the GTT, calculated as means of pre-infusion samples; metabolite concentrations at each post-infusion time point; basal NEFA:cholesterol ratio; area under the curve (AUC) for glucose and insulin, calculated as the increment above basal concentration during the 60 min following glucose infusion; clearance rate (CR) for glucose, determined assuming first order kinetics using the model Glc_t _= Glc_peak _× e^-tCR^, including post-infusion peak of glucose concentration (Glc_peak_) and concentration at progressive time points (Glc_t_); maximum increase for glucose and insulin, calculated as the difference between the basal concentration and the highest concentration. Comparisons of described variables of the breeds', except blood metabolites measured repeatedly, were performed according to a model considering discrete effects of breed and farm. To test the differences in blood metabolite concentrations between the breeds at different time points, and between selected time points within breeds, the proper contrasts were defined following the model:

This model takes into account discrete effects of breed (breed_i_), farm (farm_j_), time point (time_k_), breed and time point interaction and residual error (ε_ijkl_) with the nonzero covariance between the error terms corresponding to the observations of the same cow. The farm and time interaction effect and the linear effect of time from parturition to GTT were not statistically significant for any of the measured parameters and were omitted from the model. The characteristics of blood metabolites; BCS at calving, at GTT and BC loss; cows BW and ECM yield, and blood NEFA:cholesterol ratio during the GTT were compared according to the model, considering discrete effects of breed and farm. The models were analysed using the MIXED Procedure with the SAS System (version 9.1.3; SAS Institute Inc., Cary, NC).

Values given hereinafter are least square means presented with standard errors. Significance has been declared as follows: significant (*P *≤ 0.01 or *P *≤ 0.05), tendency (*P *≤ 0.1) and not significant (*P *> 0.1). The correction for multiple testing was made by the Bonferroni-Holm method.

## Results and discussion

**Figure 1:**
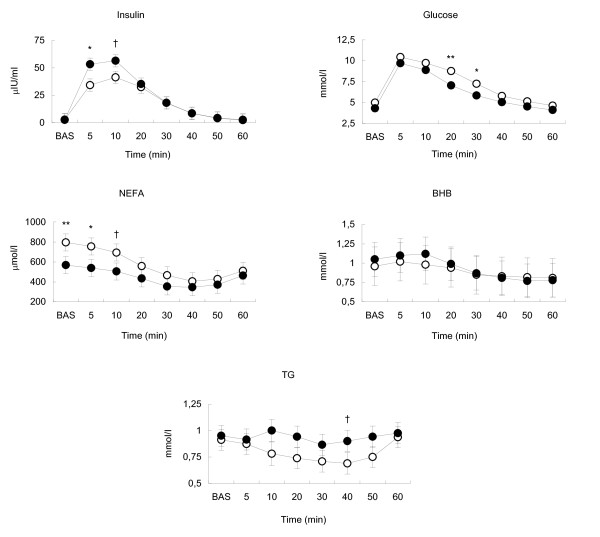
**Least square means with standard errors of basal concentration (BAS) and dynamics during the glucose tolerance test (GTT) for blood insulin, glucose, non-esterified fatty acids (NEFA),, β-hydroxybutyrate (BHB) and triglycerides (TG) in Estonian Holstein (EH - empty circle) and Estonian Red (ER - solid circle) cows**. Asterisks above the data points indicate significant differences between the breeds (** - *P *≤ 0.01, * - *P *≤ 0.05) or tendency to differ († - *P *≤ 0.1).

In this study we compared glucose-induced blood insulin and metabolite responses in two breeds one month after parturition. As reported previously, *post partum *BC loss and blood NEFA concentration correlate negatively with cows' energy balance [7]; the energy balance in our study was assessed using these indirect measures. BCS was lower in EH compared to ER at calving (3.27 ± 0.12 and 3.50 ± 0.12, *P *< 0.05) and at the time of the GTT (2.71 ± 0.11 and 3.02 ± 0.11, *P *< 0.05), while BC loss did not differ between breeds thus indicating that there was no difference in energy balance between the breeds. BCS decreased during the seven weeks *post partum *in both breeds, indicating that cows were in NEB during the GTT. On the other hand, blood NEFA basal concentration at the time of the GTT was higher in EH compared to ER (795 ± 85 μmol/l in EH and 569 ± 85 μmol/l in ER, *P *< 0.01; Figure 1), suggesting more intensive lipolysis, along with deeper NEB, at the time of the GTT in this breed.

There was a time effect on blood insulin concentration (*P *< 0.01); in response to glucose infusion insulin concentration in blood increased (*P *< 0.01) from basal level (2.8 ± 5.6 μIU/ml in EH and 2.5 ± 5.6 μIU/ml in ER) by the 5^th ^min postinfusion. At this time point it was lower in EH compared to ER (34.2 ± 5.6 μIU/ml in EH and 53.3 ± 5.6 μIU/ml in ER, *P *< 0.05). Blood insulin reached peak level, which tended to be lower in EH compared to ER (41.3 ± 5.6 μIU/ml in EH and 56.5 ± 5.6 μIU/ml in ER, *P *< 0.1), at 10 min after infusion (Figure 1). This was followed by a decrease to the pre-infusion level by the 60^th ^min (*P *< 0.01; Figure 1). There were no significant differences between the breeds' blood insulin maximum increase and AUC (Table 2). After performing a Bonferroni-Holm correction the overall time effect as well as the increase of blood insulin from basal level to peak and following decrease to initial level remained significant in both breeds, while significant differences between the breeds disappeared. In both breeds glucose infusion led to an increase in blood glucose concentration from the basal level (4.99 ± 0.40 mmol/l in EH and 4.30 ± 0.40 mmol/l in ER) to the peak (10.43 ± 0.41 mmol/l in EH and 9.70 ± 0.40 mmol/l in ER) 5 min after infusion (*P *< 0.01). This was followed by a decrease to the pre-infusion level by the 60^th ^min (*P *< 0.01; Figure 1), which represented a time effect (*P *< 0.01). There was also tendency for a breed effect (*P *< 0.1) on blood glucose concentration; blood glucose was higher in EH compared to ER 20 min (*P *< 0.01) and 30 min (*P *< 0.05) after infusion (Figure 1). There were no significant differences in blood glucose maximum increase, CR and AUC between the breeds (Table 2). Similarly to insulin the changes in blood glucose concentration within the breeds as well, as the overall time effect, remained significant after Bonferroni-Holm correction, while differences between the breeds persisted only at 20 min. General dynamics of blood insulin and glucose during the GTT are in line with previous data [17,18,22]. In our study EH cows had higher NEFA levels in blood plasma indicating a more intense lipolysis compared to ER cows. According to Hammon et al. [12] reduced insulin secretion is associated with elongated glucose half-life and with higher milk yield at the time of the GTT in Holstein × Charolais F2 crossbreed cows 30 days *post partum*. In our study ECM yields between the breeds did not differ significantly.

**Table 2:** Least square means of glucose tolerance test (GTT) measurements for blood glucose and insulin in Estonian Holstein (EH) and Estonian Red (ER) cows.

	Breed			
			
Parameter	EH	ER	SE^†^	P
Insulin				

Maximum increase relative to basal concentration; μIU/ml	39.3	57.8	9.9	0.19

Area under the curve (AUC); μIU/ml × min	875.3	1119.3	220.3	0.44

Glucose				

Maximum increase relative to basal concentration; mmol/l	5.72	5.54	0.39	0.76

Area under the curve (AUC); mmol/l × min	122.87	104.15	10.41	0.22

Clearance rate (CR); %/min	1.42	1.52	0.10	0.48

† Due to the equal number of cows in breed groups the standard errors of least square means are the same for both breeds and only one value is shown

However, during the GTT blood insulin remained lower in EH compared to ER at 5 and 10 min, which was accompanied with higher blood glucose at 20 and 30 min (Figure 1) indicating less efficacious uptake of glucose by peripheral tissues in EH. At the same time, in the lactating cow a large proportion of glucose is taken up independently of insulin by the mammary gland [23]; in our study this proportion was not distinguished from insulin-dependent uptake of glucose by adipose tissue and muscle.

In adipose tissue insulin reduces lipolysis by inhibiting hormone-sensitive lipase [24]. In our study, as in previous reports [25-27], a glucose-induced increase in insulin secretion in both breeds also led to a decrease in blood NEFA from the basal level (795 ± 85 μmol/l in EH and 569 ± 85 μmol/l in ER) to a nadir (406 ± 85 μmol/l in EH; 347 ± 85 μmol/l in ER) at 40 min (*P *< 0.01) followed by an increase from this point onwards (Figure 1), which represented a time effect (*P *< 0.01). There was also a breed effect on blood NEFA concentration (*P *< 0.05): compared to ER, the EH had higher basal concentration of blood NEFA (*P *< 0.01) and higher NEFA level 5 min after glucose-infusion (*P *< 0.05); 10 min after infusion in EH NEFA tended to be higher compared to ER (*P *< 0.1; Figure 1). After Bonferroni-Holm correction the overall time effect on blood NEFA and the decrease of NEFA concentration from basal level to nadir remained significant in both breeds. Significant differences between the breeds disappeared; although a tendency to differ persisted for basal concentrations of blood NEFA. Observed blood NEFA dynamics in breeds possibly indicates a higher tendency towards basal lipolysis in adipose tissue in EH compared to ER. At the same time, on the basis of these results it remains unclear why the differences in blood NEFA concentrations between the breeds decreased during the GTT.

As the result of insulin-mediated inhibition of lipolysis and ketogenesis, blood BHB concentration decreased during the GTT in EH (*P *< 0.01) and ER cows (*P *< 0.01; Figure 1) with a time effect (*P *< 0.01). There was no significant difference between the breeds. Observed decrease remained significant after Bonferroni-Holm correction.

Blood TG basal concentration did not differ between the breeds (0.91 ± 0.10 mmol/l in EH and 0.95 ± 0.10 mmol/l in ER). At the same time, during the GTT, ER cows' blood TG concentration was largely unchanged, while in EH cows there was a decrease from basal level to nadir 40 min after glucose infusion (*P *< 0.01), there being a tendency to breed differences at this time point (0.69 ± 0.10 mmol/l in EH and 0.90 ± 0.10 mmol/l in ER, *P *= 0.1), followed by an increase to the 60^th ^min (*P *< 0.01), revealing a time effect (*P *< 0.01; Figure 1). When adjusted by the Bonferroni-Holm correction, the tendency to breed differences at 40 min disappeared, while time effect on blood TG as well as changes in blood TG concentration within EH remained significant. In adipose tissue of non-ruminant species insulin has a stimulating effect on the activity of lipoprotein-lipase [24,28], the enzyme hydrolysing TG within the complex of very low density lipoproteins, increasing uptake of fatty acids by adipocytes along with plasma triglyceride clearance [29]. As with adipose tissue, insulin has a stimulating effect on lipogenic enzymes in the liver [24,30]. We suggest, that during the GTT in ER cows the balance between insulin-stimulated plasma TG clearance and resynthesized TG release from the liver persisted but in EH cows it did not. As there is no evidence to support the idea of enhanced TG clearance in EH cows during the GGT we consider reduced insulin sensitivity of the liver explains the change in blood TG balance in EH. This proposal of a possible decrease in hepatic lipogenesis of EH cows is supported by the following. First, the higher blood basal NEFA concentration in EH compared to ER (*P *< 0.01) could play a role. As stated previously, raised levels of blood NEFA may adversely influence insulin signalling in the liver [24], suppressing the lipogenic effect of insulin. Secondly, there is evidence of a relationship between hepatic lipidosis and reduced insulin sensitivity [31]; in addition, hepatic lipidosis is related to decreased packaging and secretion of triglycerides in the liver [32].

According to Holtenius [33] a raised blood NEFA:cholesterol ratio is considered to be a marker of hepatic lipidosis. In our study EH cows had higher NEFA:cholesterol ratios (3.4 ± 0.4 in EH and 2.2 ± 0.2 in ER, *P *< 0.05) compared to ER, suggesting the possibility of a greater expression of hepatic lipidosis in EH cows. Therefore we suggest that the decrease in blood TG concentration during the GTT could be attributed to less intensive resynthesis and release of TG from the liver of the EH cows.

## Conclusion

In our study less pronounced glucose-induced increase in blood insulin concentration was accompanied by higher blood glucose concentrations in EH compared to ER cows. In response to glucose-induced insulin secretion lipolysis in adipose tissue and ketogenesis in the liver were reduced during the GTT in both breeds. Blood TG concentration in EH during the GTT decreased, while in ER the TG level remained largely unchanged.

## Abbreviations

AUC: area under the curve; BCS: body condition score; BHB: β-hydroxybutyrate; BW: body weight; CR: clearance rate; DIM: days in milk; DM: dry matter; DMI: dry matter intake; ECM: energy corrected milk; EH: Estonian Holstein; ER: Estonian Red; GTT: glucose tolerance test; ME: metabolizable energy; MP: metabolizable protein; NEB: negative energy balance; NEFA: non-esterified fatty acids; TG: triglycerides; TMR: total mixed ration.

## Competing interests

The authors declare that they have no competing interests.

## Authors' contributions

All authors participated in designing the study, interpreting the data and compiling the manuscript. HJ carried out the study, compiled the data and drafted the manuscript. KL helped to draft the manuscript. JS and AI carried out the study, TK performed the statistical analyses and OK coordinated the study. All authors read and approved the final manuscript.
